# A Benchmark Evaluation of Adaptive Image Compression for Multi Picture Object Stereoscopic Images

**DOI:** 10.3390/jimaging7080160

**Published:** 2021-08-23

**Authors:** Alessandro Ortis, Marco Grisanti, Francesco Rundo, Sebastiano Battiato

**Affiliations:** 1 Department of Mathematics and Computer Science, University of Catania, 95125 Catania, Italy; marco.grisanti@phd.unict.it (M.G.); battiato@dmi.unict.it (S.B.); 2 STMicroelectronics, ADG Group—Central R&D, 95121 Catania, Italy; francesco.rundo@st.com

**Keywords:** stereoscopy, stereoscopic image compression, multi-picture object, image encoding

## Abstract

A stereopair consists of two pictures related to the same subject taken by two different points of view. Since the two images contain a high amount of redundant information, new compression approaches and data formats are continuously proposed, which aim to reduce the space needed to store a stereoscopic image while preserving its quality. A standard for multi-picture image encoding is represented by the MPO format (Multi-Picture Object). The classic stereoscopic image compression approaches compute a disparity map between the two views, which is stored with one of the two views together with a residual image. An alternative approach, named adaptive stereoscopic image compression, encodes just the two views independently with different quality factors. Then, the redundancy between the two views is exploited to enhance the low quality image. In this paper, the problem of stereoscopic image compression is presented, with a focus on the adaptive stereoscopic compression approach, which allows us to obtain a standardized format of the compressed data. The paper presents a benchmark evaluation on large and standardized datasets including 60 stereopairs that differ by resolution and acquisition technique. The method is evaluated by varying the amount of compression, as well as the matching and optimization methods resulting in 16 different settings. The adaptive approach is also compared with other MPO-compliant methods. The paper also presents an Human Visual System (HVS)-based assessment experiment which involved 116 people in order to verify the perceived quality of the decoded images.

## 1. Introduction

A stereoscopic image, or stereopair, is composed by a pair of images, named left and right views, taken at the same time on the same scene by two cameras from different points of view. The acquisition process aims to emulate the binocular view of the Human Visual System (HVS). In this sense, the distance between the two cameras is set to the distance between the human eyes’ pupils. During the presentation of such images, proper filters and devices are used to let the left eye see only the left image, and the right eye see only the right image. In this way, the viewer has the perception of a real 3D scene, as the two single views presented to the HVS are merged in the brain visual cortex.

The storage requirement for stereoscopic images is tat least twice that of to the storage needed for a single image, hence the motivation for the study of stereoscopic compression techniques applications. The Multi Picture Object (MPO) is a standardized file format used to encode the Multi Picture Format for multi-view images defined by the Consumer & Imaging Products Association (CIPA) [1]. Such a format implements a chain of JPEG-encoded images properly embodied in a unique file (i.e., the MPO file) with a set of additional information useful to recover the single images and correlate them one each other in the context of the multi-view acquisition. In the specific case of stereoscopic images, only two images are encoded in the MPO file. However, the format allows the encoding of an unlimited number of views. Furthermore, the data needed to reconstruct the multi-view image from an MPO file are stored in the meta-data of the image, exploiting the JPEG metadata fields of the first image. As a consequence, the MPO format does not require additional fields or the change of data format.

The JPEG pipeline [2] and its extensions [3] represent a standard for the compression of digital images, by allowing an optimized encoding of the image palette considering both quality and memory factors. Based on the JPEG encoding process, several works tried to optimize one or more elements of the pipeline. For instance, [4] proposed an alternative strategy to devise proper quantization tables. The experiments showed that the method was able to improve the compression performance of established JPEG compression schemes [2,5].

MPO provides a standard for multi-view images, bringing several practical advantages. However, most of the state of the art works that address the problem of stereo image compression implement encoding techniques that do not take into account the standardization of the compressed images.

Most of the existing approaches store one of the two views, the disparity map and a residual version of the other view [6,7,8]. Then, the residual image and the disparity map are used to restore the compressed view, with a certain degree of confidence. This approach needs a method to store the additional data, such as the disparity map and the residual image. In the majority of cases, an entropy-based coding is employed for the disparity map, whereas Discrete Cosine Transform (DCT) is used for the residual image. In particular, the work in [8] proposes the estimation of the stereoscopic disparity map from the local 1D-Fast Fourier Transform (FFT) computed on the left and the right image. Approaches based on the disparity map pose problems in the reconstruction. In particular, although gradual transition is usually observed in disparity maps, object edges may produce abrupt change of the parameter. This will cause two main issues: pixels without any assignment (i.e., falling disparity), and matching problems due to double assignments [8]. For this reason, in addition to the left image, the right image and the disparity map, the approach in [8] defines an error image to the encoding pipeline of a stereopair.

Schenkel et al. [9] proposed a joint decoding approach of the two views for the compression of a stereopair. The proposed method then performs an enhancement of the image pairs, previously compressed using the JPEG pipeline. However, some areas of the images cannot be reconstructed. Moreover, the experiments shown that with middle values of the JPEG quality compression, decreases in terms of Peak Signal to Noise Ratio (PSNR) are observed, and some ghosting artefacts appear.

The work in [10] proposes a variable size-block coding algorithm for stereoscopic images that jointly optimizes the block sizes and the quality of the disparity map computed from the compressed images. In particular, the system applies a fine-grained pixel blocking on the image areas with more detail, used to encode the disparity map. This approach has been designed with the aim to obtain an high quality of the reconstruction while reducing the bit-rate of the stereopair. Although the experimental results achieved in [10] are promising, it requires an overhead of computational effort due to the block layout definition for the encoding, as well as additional data consisting of the tree structure describing the blocking layout, the block-length map and other information needed for the stereo pair decoding. All these data structures need to be stored properly with an ad hoc file format, besides the image payload.

In [11], Poolakkachalil et al. presented an approach for symmetric stereoscopic image compression followed by an arithmetic coding named Stereoscopic Image Compression using Curvelet and Arithmetic Coding (SICCAC), which is mostly based on the still image compression method proposed in [12], that applies a curvelet transform for image encoding. This work has been further extended in [13], which proposes an encoding pipeline in which the difference and the average of the two stereo images are quantized and then encoded exploiting an adaptive arithmetic coding (S2ICAC). Other methods exploit entropy-based coding to encode the stereoscopic images, such the method evaluated in [14], also known as Stereoscopic Image Compression using Huffman Coding (SICHC).

Most of the above mentioned works assume that the stereopair has been acquired using a parallel camera optical axis stereo system, where the convergent axis setting is ignored. The camera optical axis convergence involves a perspective effect that generates a vertical parallax. With parallel camera optical axis the vertical parallax is removed. Approaches that try to completely reconstruct one view from the other views do not take into account that a perfect reconstruction is not possibles, as discussed in [8]. Moreover, in real scenes, there are areas on each image that are not visible in the other image. When the problem of stereoscopic image compression is considered in the context of real and high quality images, approaches that ignore parts of the scenes cannot be considered; issues related to standardization of the storage (i.e., file) and transmission formats also have to be addressed.

The work in [15] introduced an end-to-end deep neural network for stereoscopic image compression. In particular, the homography *H* between the left and right image is estimated, then the left image is mapped to the right view by exploiting *H* and the residual image is stored. The proposed architecture includes a deep regression model to estimate the homography matrix, two autoencoders, and cross-quality enhancement (CQE) network. Although the method in [15] achieves interesting results, it requires very high resources, in terms of data quality and quantity, computational time, and specific hardware. Moreover, it forces the input images to a specific resolution as well as introducing a bias related to the training datasets, which are divided among close views (InStereo2K) [16] and far-views (KITTI) [17] stereopairs. In particular, the method in [15] exploits a deep neural network to estimate the *H* matrix, which could be simply inferred by using traditional geometry-based methods which only require a few correspondences between the left and right views. During encoding, the left image is geometrically transformed by means of *H* and then the two images are further processed through two separate autoencoders. Such a processing breaks the geometric constrains which are then inferred during the encoding step. For this reason, the CQE network is needed. The authors proposed two deep models, the first requires 50.5 M parameters and the second 69.3 M. The models have been trained on two datasets of high quality stereoscopic images [16,17] including about 2000 examples each.

The authors of [18] presented a preliminary experiment on the exploitation of stereoscopic image redundancy to reduce the bitrate of stereopairs. In particular, the paper combines two algorithms. One performs better at low and mid-range bitrates, the other at mid and high-range bitrates. One iteratively modifies the disparity map to improve the bitrate-distortion trade-off using a Lagrangian multiplier. The other selects each disparity on a block basis, according to a simplified model of how JPEG deals with the compensation refinement. However, the main contribute of [18] is theoretical, indeed the proposed approach has only been tested on two stereoscopic images.

**Figure 1:**
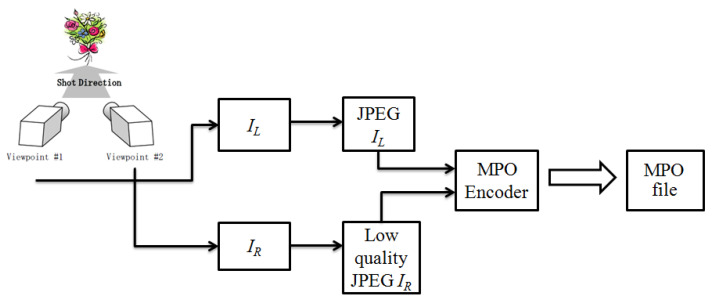
Encoding pipeline. Each view is coded according to the MPO format (i.e., by applying the JPEG compression) using different quality factors.

In [19], the authors presented a strategy for MPO image compression that significantly reduces the space needed to store a stereopair with very low quality loss. One of the main advantage of this method is that the compression phase allows us to obtain an MPO compliant compressed file (see Figure 1). The decoding phase, through a proper restoration phase, reconstructs the original information after the MPO standard decoding of the two images. The paper in [20] presents an improvement of the work in [19] drastically reducing the overall reconstruction phase complexity, while keeping the same reconstruction quality. Compared with respect to previous methods, the methods in [19,20] are explicitly designed for the MPO format, formalizing a proper coding/decoding pipeline that can be implemented directly on acquisition devices. Therefore they support the standardization, and work indifferently on stereoscopic images acquired using both a parallel or a convergent stereo camera system. However, the experiments have been performed on a reduced number (i.e., 23) of selected stereopairs. The two methods achieved the same performances in terms of reconstruction quality; however, the approach presented in [20] drastically improves the method in [19] in terms of computational costs.

In this paper, we perform an extended benchmark evaluation of [20] on a large set of images taken from standard and well-known datasets designed for evaluations of algorithms on stereoscopic images in real scenarios. In addition, we present the results of a subjective assessment of the reconstructed image quality, which involved 116 participants who evaluated 10 images each, producing a set of 1160 HSV-based tests on randomly selected images from the considered datasets. Then, we also evaluated the method in [20] on the dataset used in [11,13].

The remainder of the paper is structured as follows. Section 2 clearly states the motivations of the presented benchmarking, by comparing pros and cons of the state-of-the-art methods. The employed coding/decoding pipeline is detailed in Section 3, comparing the differences between the approaches proposed in [19,20]. The two approaches are explained in detail and compared in terms of computational complexity. Section 4 presents an experimental evaluation of the adaptive stereoscopic image compression approach, considering 16 different experimental settings on 60 stereopairs taken from public standardized datasets with different resolutions and acquisition settings (i.e., parallel or convergent cameras). It also presents a comparative evaluation with other MPO-compliant methods, as well as an HVS-based experiment aimed to evaluate the perceived quality of the reconstructed images. Section 5 concludes the paper.

## 2. Motivations

In the context of multi-view pictures, the MPO format is an established standard by years, currently adopted for storing stereoscopic and 3D pictures taken with a photo camera equipped with multiple lens system by several device producers, including game consoles. In this context, the methods proposed in [11,13,19,20] define encoding/decoding pipelines which maintain the compatibility with MPO format and are independent from the acquisition settings (i.e., parallel/convergent axis, image resolution, etc.). As discussed in the previous Section, other methods often require additional payload to store the stereopair and ad hoc data format [6,7,8,9], or present ghosting artefacts [9]. Standardization of image encoding/decoding pipelines brings several advantages, especially when such methods are embedded on the acquisition/rendering devices, requiring high performances with limited resources. Methods based on very Deep Neural Networks (DNN) requiring millions of parameters and complex non-linear operations cannot be embedded on such devices [15]. Moreover, DNNs have millions of parameters, each with complex inter-relationships. In this way, Deep Learning models have been criticised to be a black-box, in contrast with deterministic and explainable geometrical based approaches which have full transparency and allow one to directly observe whether the achieved solution will work outside of a training environment [21]. Indeed, traditional Computer Vision techniques are often preferred over DNNs in a range of applications from reducing training time, processing and data requirements, in particular to be applied on geometrical related fields [21] (e.g., structure from motion, Panoramic-stitching, etc.). The method assessed by our benchmarking evaluation has been specifically designed for MPO images, as well as the methods considered for the comparative evaluation. All the compared approaches are MPO-compliant and can be easily embedded within low-resource devices as they re-design the encoding-decoding pipeline already implemented in such devices. Moreover, these methods are independent from the stereo acquisition settings (i.e., parallel or converging camera axis). However, the experiments presented so far were limited in quantity, quality, and variability of the images. Moreover, no comparison with respect to other methods were presented, as well as a subjective assessment of the perceived image quality. Given the above, we realized need for a standardized, large-scale, benchmark evaluation.

The benchmark evaluation presented in this paper includes:

- Experiments on 60 stereopairs of the Middlebury-scenes datasets including versions between 2001 and 2014;
- Images which resolution variates from 375 × 450 to 2016 × 2960;
- A total of 16 different evaluation settings, by combining different feature detection and geometry estimation;
- Comparative evaluation with other five methods published more recently, taking into account both bitrate saving and reconstruction quality, on the same publicly available dataset;
- Subjective assessment conducted with high number of tests and high participant population variability confirmed that the reconstructed image is indistinguishable from the high quality one.

## 3. Evaluated Pipeline

The following paragraphs present the encoding/decoding strategy. In particular, the two different matching approaches implemented in [19,20] are described in detail and compared in terms of computational complexity.

### 3.1. Encoding Pipeline: Asymmetric Compression

In the encoding phase, the proposed method encodes one image view with a low JPEG quality factor [2]. During encoding, the redundancy between the two images is exploited to enhance the low quality image by using the high quality one as reference. The enhancing process compares image blocks properly extracted from the two views. In the image areas in which no a reliable level of redundancy is detected (i.e., high difference between low and high quality images), only the information from the low quality patch is considered. As a consequence, there is a certain level of lossy on the resulting enhanced view. However, experiments show that this losing rate is numerically negligible and not perceptible visually. The JPEG encoding pipeline defines a quality level to control the amount of compression. A low-quality image results in a smaller JPEG file, whereas a high-quality image produces a relatively large file. The quality level determines the quantization tables used during the JPEG encoding pipeline, these tables control the amount of loss during the compression and hence the size of the generated file. Therefore, the quality level directly affects the visual quality of the image and the file size.

The amount of JPEG compression is typically measured as a percentage of the quality level. In general, quality levels of 90% or higher are considered high quality images, 80–90% is medium quality, and 70–80% is low quality. Images compressed with quality values below 70% are typically a very low quality. With such quality levels, edges are no longer sharp and compression artefacts are visible. For these reasons in our experiments we compressed the low quality image considering quality levels equal to 70% and 65%.

Figure 1 details the proposed encoding pipeline in which the two image views are encoded differently. Each image block of the low quality view $I_{R}$ is then reconstructed by exploiting the high quality view $I_{L}$. In particular, we conventionally encoded the left image $I_{L}$ with an high JPEG quality rate and the right image $I_{R}$ with a low quality rate, but the same pipeline can be applied by inverting the role of the right and left views.

### 3.2. NCC-Based Decoding Approach

The approach proposed in [19] implements a image blocks matching method based on the correlation between image patches. A common way to match a given pattern *t* within an image *I* is to consider the Normalized Cross Correlation (NCC) score ncc(u,v) computed at each possible position (u,v) of the template *t*, which has been shifted by *u* and *v* steps in the *x* and *y* direction, respectively. The NCC coefficients are defined as follows:

(1) $$ncc\left(u,v\right)=\frac{\sum_{x,y}\left\{\left[I\left(x,y\right)-{\bar{I}}_{u,v}\right)\right]\left[t\left(x-u,y-v\right)-\bar{t}\right]\}}{{\left\{\sum_{x,y}\left[I\left(x,y\right)-{\bar{I}}_{u,v}\right)\right]}^{2}\sum_{x,y}{\left[t\left(x-u,y-v\right)-\bar{t}\right]}^{2}{\}}^{0.5}}$$

where ${\bar{I}}_{u,v}$ is the mean value of the pixels I(x,y) located within the area of the template *t* shifted by (u,v), and $\bar{t}$ is the average value of the pixels of *t*.

At decoding time, both right and left views are subdivided into not overlapping patches of size N×M×3. For each block, the decoding procedure exploits the redundancy between blocks extracted from the left and the right image with the aim to enhance the quality of the blocks extracted from the low quality one. The decoding algorithm is applied on each block extracted from each channel.

#### Matching Approach

Given the generic $i^{t}h$ extracted from the low quality image $I_{R}$, named $b_{i}^{R}$, the objective is to find the best sub-image of the high quality image $I_{L}$, which redundancy can be exploited to enhance $b_{i}^{R}$. To this aim, the algorithm considers two candidate blocks:

- The N×M block of $I_{L}$ which is is located at the same position of $b_{i}^{R}$;
- The N×M block of $I_{L}$ obtained by computing the Normalized Cross Correlation (NCC) [22] between $b_{i}^{R}$ all the N×M sub-images of $I_{L}$ and considering the sub-image with the highest NCC value.

In practice it is not needed to compute the NCC for every possible position of $b_{i}^{R}$ in $I_{L}$. An optimized approach would compute the NCC in a restricted area of $I_{L}$ taking into account the original position of $b_{i}^{R}$.

The procedures then select the candidate block which minimizes the Sum of Absolute Differences (SAD) with $b_{i}^{R}$. The selected block is then exploited by the enhancing procedure described in Section 3.4.

### 3.3. Geometry-Based Decoding Approach

The approach presented in [20] extends the work in [19] by focusing on the improvement of the matching phase efficacy and the optimization of its computational costs. These two objectives have been obtained by leveraging on the geometric constrains of a stereoscopic pair.

#### 3.3.1. Epipolar Geometry

Epipolar geometry describes the properties and the geometrical relationships between two images that describe the same 3D scene in a stereoscopic image. Such geometry is independent of scene structure, as it is fully described by the parameters of the two cameras and their relative locations [23].

**Definition 1** (Fundamental Matrix)**.** *The fundamental matrix, denoted as F, is a 3×3 matrix of rank 2 that, for any pair of corresponding points $x_{1}$ and $x_{2}$ satisfies the following condition:*

(2) $$x_{1}^{\prime }Fx_{2}=0$$

*where $x_{1}$ is a point of one view which corresponds to $x_{2}$ in the other image view.*

The fundamental matrix encodes the intrinsic geometry of the acquisition setting (i.e., the relative position and orientation of the two cameras) with respect to the scene. Indeed, $x_{1}$ and $x_{2}$ in Equation (2) correspond to the projection of the same real 3D point on the two camera image planes. Therefore, given two points taken from the left and right views of a stereoscopic image depict the same real-world point, they must satisfy the relationship in Equation (2). This property has been exploited by the method proposed in [20] to drastically reduce the search range of correspondences between the left and the right views. Hence, to reduce the computational cost during the matching phases. Indeed, according to the point line dualism theorem, given a stereoscopic image, for any point $x_{1}$ in the first view, there exists a corresponding epipolar line $l_{2}$ on the second view. Moreover, any point $x_{2}$ in the second view matching $x_{1}$ lies on $l_{2}$. Indeed, $l_{2}$ is the projection of the ray from the point $x_{1}$ to the second view, which passes through the first camera’s centre.

We can map $x_{1}$ to its corresponding epipolar line $l_{2}$ on the second image by exploiting the following Equation:

(3) $$l_{2}=Fx_{1}.$$

In other words, the fundamental matrix F allow us to have a direct relationship between a given point $x_{1}$ in the first image, and its corresponding epipolar line $l_{2}$ in the second view, which contains the corresponding point $x_{2}$. Therefore, the search of the point $x_{2}$ can be limited to the points in $l_{2}$.

#### 3.3.2. Image Blocking

In the decoding approach described in Section 3.2, the low quality view is subdivided into a number of not overlapping blocks. The block size is selected adaptively depending on the input image dimensions with the aim to cover all the image area, resulting in a few number of rather big blocks. With such block dimensions, each image is subdivided in about 12 blocks. Such a low number of blocks allows the execution of the decoding approach in a reasonable time. Then each block extracted from the low quality image is compared with the high quality one using a template matching approach based on the NCC computation to detect the most similar high quality area.

This approach presents several limits. Due to the high dimensions of the blocks, each extracted image patches depict a rather big area of the scene (e.g., with 12 blocks in total, each block includes more than the 8% of the image), including different objects and several details of the pictured scene. Due to the differences between the point of views of the two cameras, each object appears slightly different in the resulting left and right views. Such differences augment with the dimensions of the extracted blocks, as more objects are included in the corresponding patches, especially in cluttered scenes. Moreover, the procedure is repeated for each image channel. As a consequence, the matching procedure results coarse and computationally expensive.

**Figure 2:**
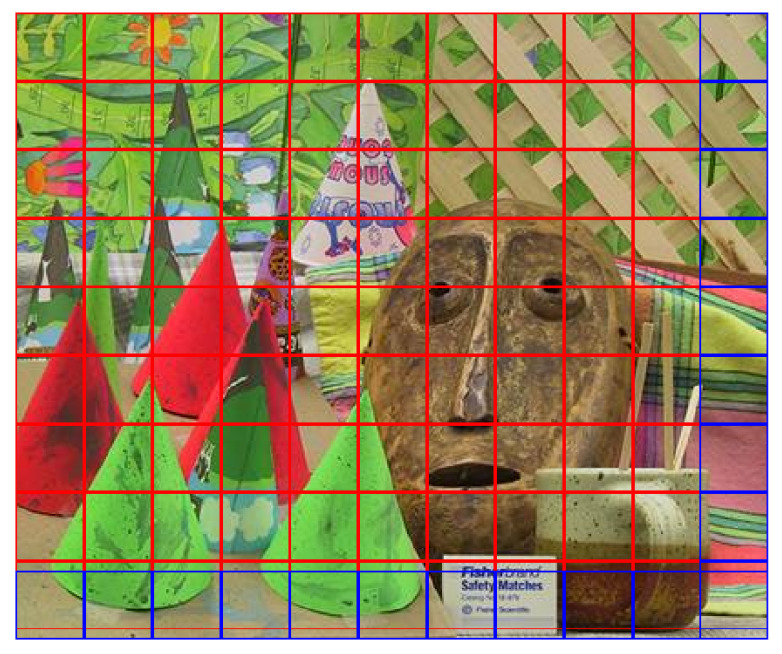
Example of blocking schema employed in [20].

In contrast, the method presented in [20] subdivides the images considering very few blocks. All blocks have the same dimensions and the matching procedure is applied simultaneously to all the colour channels of the block. In particular, a radius *r* is set to a constant value (r=20 in [20]), then the low quality image is partitioned into (r+1)×(r+1) not overlapping blocks. If needed, to cover all the image area, some overlapping blocks are defined in the right and in the bottom part of the image. An example of block definition is shown in Figure 2. In this example, the image “Cones” of the Middlebury 2003 dataset [24] is subdivided into 110 blocks, including a number of overlapping blocks (depicted in blue). Reducing the dimensions of the image blocks allows a fine-grained search of the processed data, thus avoiding working with big matrices of coarse pieces of the scene, where several objects’ positions with respect to the camera system can be very different. As an example, considering the same image, the method used in [19] defines 12 blocks with size 360×360, whereas the method in [20] extracts 972 blocks and is computationally more efficient.

#### 3.3.3. Matching Approach

The main improvement of the geometry based approach is related to the matching strategy. In the following, we conventionally consider the left view as the high quality image, whereas the right view is considered as the low quality one. Given a block $b_{i}$, extracted from the right view, the system considers the block’s centre $c_{i}$ and computes the corresponding epipolar line $l_{i}$ on the left view by applying the point-line relationship defined by Equation (3). According to the epipolar geometry (see Section 3.3.1), we known that the unknown point that corresponds to $c_{i}$ on the left image lies on $l_{i}$. Therefore we can limit the matching of the whole block $b_{i}$ to the possible positions of $b_{i}$ on the left image obtained by shifting the block centre $c_{i}$ along the epipolar line $l_{i}$. Therefore, the main benefit is given by the fact that the matching of the image block is limited to a set of possible positions of $c_{i}$ on the epipolar line. The number of such positions is approximately equal to the image width; however, as detailed in the following paragraphs, the employed approach further reduces the search range. The epipolar lines related to the extracted blocks’ centres are computed by exploiting the Equation (3); therefore, the estimation of the fundamental matrix *F* is needed.

The matrix *F* can be estimated starting from a number of correspondences between the left and the right views. Indeed, each pair of matching points between the two images provides a linear constrain on *F* (i.e., Equation (2)). As consequence, the fundamental matrix *F* can be estimated linearly from at least eight independent pairs applying the eight point algorithm [25]. The eight point algorithm is simple and effective; however, it is sensible to the precision of the input correspondences. An alternative is represented by the Least Meadian of Squares estimation, which is robust to the presence of wrong correspondences, but requires a number of good pairs of matching points equal or greater than 50% of the input data.

**Figure 3:**
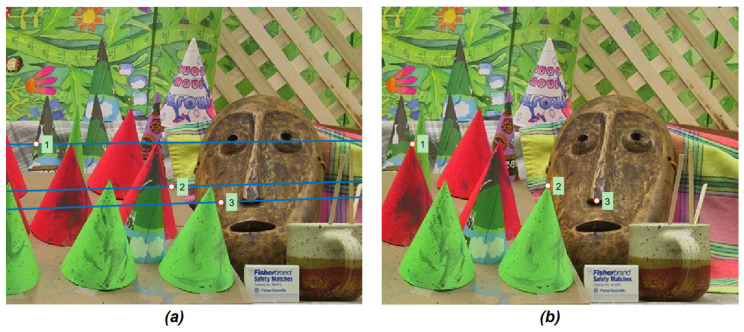
Considering any point in the right image (**b**) $I_{R}$ (1, 2, and 3), the method in [20] searches the corresponding points in the left image $I_{L}$ by computing their epipolar lines (**a**).

**Figure 4:**
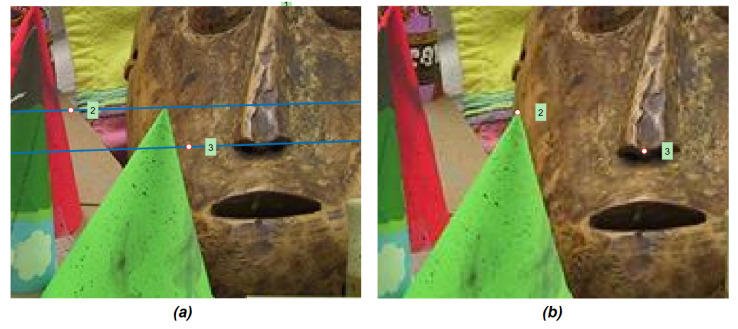
Details of the images shown in Figure 3. The tip of the cone (i.e., point number 2 in the image (**b**) depicting the right view $I_{R}$) corresponds to a point that lies on the corresponding epipolar line (i.e., line number 2 in (**a**)) on the left view $I_{L}$.

Both the eight point algorithm and the Least Meadian of Squares estimation require a number of input correspondences. These pairs of matching points have been computed considering two different approaches for local features detection, namely the SIFT (Scale-Invariant Feature Transform) [26] and Harris keypoints [27]. Figure 3 shows an example in which three epipolar lines (Figure 3a) have been computed from three sample points taken from the right image (Figure 3b) by applying Equation (3). The points drawn on the left view are the points in the epipolar line which has the same *x* of the sample points taken from the right view. Indeed, is possible to note that they do not corresponds to the three sample points of the right view. Figure 4 shows the detail of the points number two and three: these points are placed on the vertex of the cone (point two) and the tip of the masks’ nose (point three) in the right view (Figure 4b), their epipolar lines pass correctly by the corresponding points in the left image (Figure 4a). The points with the same *x* coordinates which lies on these lines (reported on the left image) appear shifted with respect to the corresponding ones. This observation suggest us that, given a point of the right view, beside from the fact that its corresponding point on the left images lies on the epipolar line (i.e., Equation (3)), the position of this unknown point is close to the point with the same *x* of the point selected in the right view.

#### 3.3.4. Range Reduction

The estimation of the epipolar line reduces the searching range to a single line. Indeed, given an image block $b_{i}$ is possible to estimate the epipolar line of its centre $c_{i}$ and perform the block matching considering only the possible patches which centre lies on $l_{i}$. Nevertheless, the number of comparisons can be further reduced by observing that:

1. The *y* coordinate of the searched point is given by the *y* values of the epipolar line;
2. The *x* coordinate of the searched point is close to the *x* of the correspondent point on the right view;
3. Equation (2) must be satisfied by any pair of corresponding points.

With the aim to minimize the searching range, the following approach has been designed in [20]. Given a point $x_{1}$, centre of a block extracted from the right view, the corresponding epipolar line $l_{1}$ is computed by exploiting Equation (3). Then, for each point $x_{2}$ ∈ $l_{1}$, the value of $x_{1}^{\prime }Fx_{2}$ is computed. Equation (2) states that if the corresponding point of $x_{1}$ is represented by the left view, the values of $x_{1}^{\prime }Fx_{2}$ represents a zero crossing line. Furthermore, according to (1), the corresponding point of $x_{1}$ lies around the point where the the value of $x_{1}^{\prime }Fx_{2}$ is zero. In order to further focus the matching search to this region, the following function is defined:

(4) $$\phi ={\left(x_{1}^{\prime }Fx_{2}\right)}^{2}$$

**Figure 5:**
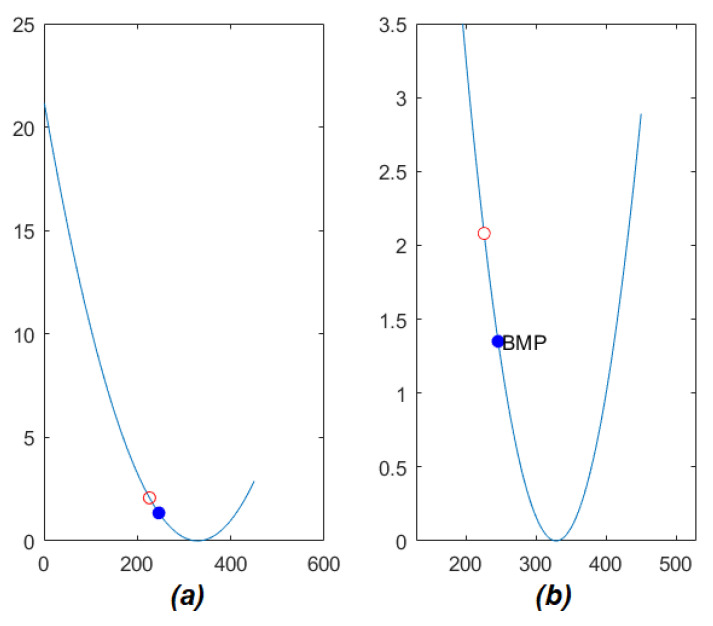
Example of matching by using the approach in [20] (**a**) and detail near the vertex of the ϕ function (see Equation (4)). The red circle is the point on ϕ with the same *x* as the point on the (**b**). The blue point represents the value of ϕ corresponding to the Best Matching Point (BMP) according to the algorithm.

The function ϕ defines a parabola. In particular, the vertex of ϕ is close to zero. An example of ϕ is shown in Figure 5. Is possible to observe how the value of ϕ grows rapidly with the distance from its vertex. Moreover, the point with the same *x* coordinate of $c_{i}$ (represented by a red circle in Figure 5) is placed nearby the vertex.

The reduction of the matching search range is finally obtained by applying a threshold to ϕ. In the experiments, it has been observed that the value 2.3 permitted to drastically remove the number of candidate blocks (see Figure 5). However, instead of using a fixed threshold heuristic, an adaptive method has been applied. It is based on the following adaptive rules:

1. The threshold value is set to 2.3;
2. If the point with the same *x* as $c_{i}$ is not included by the range obtained after the thresholding, according to the previous considerations, the threshold is augmented until this point is included in the search range;
3. If the search range is empty, the threshold is augmented iteratively by 0.5 until the range is not empty.

These rules allowed the definition of an effective adaptive thresholding strategy.

#### 3.3.5. Block Matching

Due to the approach described in Section 3.3.4, the search range is drastically reduced. Then, given a block $b_{i}$ of the right view, the matching procedure searches the best matching block of $b_{i}$ considering the candidate blocks such that the centres lie in $l_{i}$ and the *x* coordinate is in the reduced range. These constrains allow one to significantly reduce the number of comparisons between blocks. The blocks are compared by computing the Sum of Squared Differences (SSD) between $b_{i}$ and the selected candidate blocks. The block which achieves the lowest SSD is then employed during the enhancing phase (see Section 3.4).

Experiments revealed that in presence of uniform blocks the above described procedure could include false positive examples and cause the failure of the matching procedure. In these cases, if the threshold is high, the best matching block in terms of SSD could be placed far from the theoretical position computed considering the geometrical properties. In other words, if an image contains a large uniform area (e.g., a wall), the image blocks extracted from such area could match one each other because there are not enough edges that characterize the selected image patches and hence allow the SSD to catch the differences between mismatching blocks.

Considering that the JPEG compression tends to preserve low frequencies, the method in [20] discards the uniform blocks from the enhancing procedure, by filtering out the blocks with low pixel variance. This strategy allows to further reduce the number of comparisons (i.e., SSD computations), and contextually to avoid the matching issues caused by the presence of uniform candidate blocks. Experiments shown that the results are not affected by this approach in terms of quality, while time performances are significantly improved. Therefore, this simple choice allowed to maintain the quality while the matching process is further sped up.

#### 3.3.6. Partial Matching

In could happen that some areas of one image view, located nearby the border of the image, correspond to blocks of the other image view that are just partially visualized, due to the difference of the camera point of view and orientation. This happens when some parts of the scene are represented only by one of the two views. To address with the problem of partial matching, the method in [20] allows candidate blocks with lower dimensions. In particular, the procedure include all the blocks whose dimensions are equal or greater than 60% of the processed block dimensions.

To compare $b_{i}$ with partial candidate blocks, the missing pixels are filled using the values of $b_{i}$.

**Figure 6:**
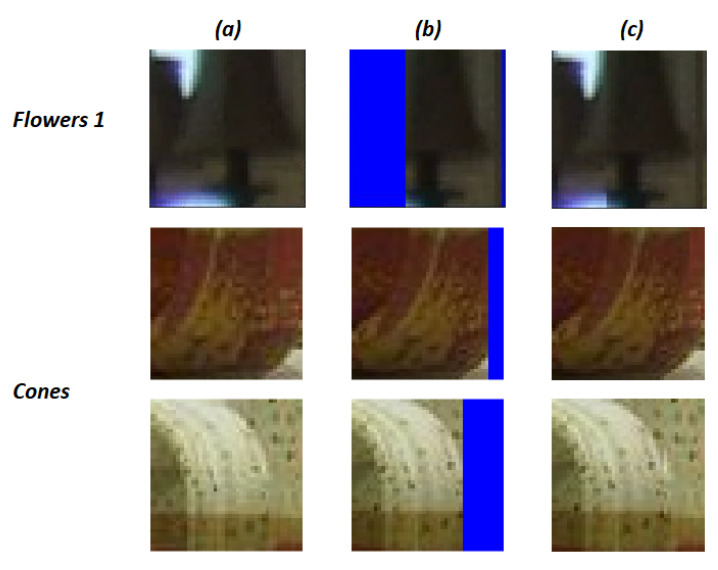
Examples of block composition performed to overcome with partial matching blocks. Each row shows an example of block composition, the first row shows the block number 111 (**a**) of the image “Flowers 1”, the partial matching block (**b**) and the composed block (**c**). The second and third rows are examples of partial matching of blocks extracted from the image “Cones”, related to the blocks number 17 and 13, respectively.

Figure 6 shows some examples of partial matching and block compositions. In particular, the first row represents an example taken from the stereopair “Flowers 1” in which the block number 111 is partially matched, whereas the second and third rows are related to the stereopair “Cones”. The first row of Figure 6 shows that a chandelier is visible in the right view of the stereopair (i.e., column (a) of the first row in Figure 6) and it is depicted in the block $b_{111}$, considering the blocking schema described in Section 3.3.2. This object is only partially depicted in the left image view. When the algorithm performs the matching procedure for the block $b_{111}$, which represents the part of the chandelier shown by both images (Figure 6a), it takes into account also a partial matching with a 41×25 block placed on the left side of the reference image. The blue area in Figure 6b represents the missing part within the image patch, which are then filled with the pixels of $b_{111}$ in the same positions, obtaining the composed block shown in Figure 6c.

### 3.4. Image Enhancing

The enhancing step employs the following equation, which is based on a simplified version of Kohonen update rule [28]:

(5) $${\bar{b_{i}}}^{R}\left(u,v\right)=\left\{\begin{array}{cc} b_{i}^{R}\left(u,v\right)+\alpha \cdot d_{i}\left(u,v\right) & if\;d_{i}\left(u,v\right)<th\\ b_{i}^{R}\left(u,v\right) & otherwhise \end{array}\right.$$

where

(6) $$d_{i}\left(u,v\right)=b_{i}^{R}\left(u,v\right)-b_{i}^{L}\left(u,v\right)$$

where ${\bar{b_{i}}}^{R}\left(u,v\right)$ is the enhanced sample, $b_{i}^{L}\left(u,v\right)$ is the sample of the block selected by the matching procedure and $b_{i}^{R}\left(u,v\right)$ is the sample which has to be enhanced. By applying Equation (5), the values of some samples of $b^{R}$ are moved closer to the corresponding values in $b^{L}$, depending of the similarity between the pair of correspondent samples. The parameters α and th (in our case alpha=0,25 and th=0043) are two coefficients that control the reconstruction procedure [28] based on the distance between the two samples values defined as $d_{i}\left(u,v\right)$ in Equation (6). The parameters in Equation (5) have been empirically obtained by grid-search evaluation previously performed [19,20] on the 23 MPO stereoscopic images from the 3DMedia collection [29]. In the extended evaluation presented in this paper we used the same parameters, which further assess the generalization capability of the evaluated approach.

### 3.5. Computational Complexity

In this Section the NCC-based matching procedure described in Section 3.2 and the geometry-based matching method described in Section 3.3 are compared from a computational point of view.

### 3.6. NCC-Based Complexity

The computational complexity of Normalized Cross Correlation (NCC) used to find matchings of a reference template with dimensions m×n within a scene image with sizes M×N is

(7) O(mnMN)

therefore, the cost of NCC-based matching, for each m×n block, is equal to

(8) $$T_{NCCmatching}=O\left(mnMN\right).$$

Let *D* be the number of blocks:

(9) $$D=\frac{MN}{mn}$$

Hence, the total cost due to the matching procedure described in Section 3.2 is

(10) $$\begin{array}{c} T_{NCCbased}=D\times T_{NCCmatching}=\\ D\times O\left(mnMN\right)=O\left(M^{2}N^{2}\right). \end{array}$$

### 3.7. Geometry-Based Complexity

The cost of the computation of the SSD between two blocks of size m×n is O(mn); therefore, the cost due to the matching method described in Section 3.3.3 is

(11) $$T_{GeometryMatching}=k\times O\left(mn\right)$$

where *k* is the number of blocks that the procedures compares to find the best matching. Thus, if *D* is the total number of processed blocks with sizes m×n, the total cost of the matching procedure of the geometry based approach described in Section 3.3 is

(12) $$\begin{array}{c} T_{GeometryBased}=D\times T_{GeometryMatching}=\\ D\times k\times O(mnMN). \end{array}$$

The value of *k* is very low due to the range reduction approach described in Section 3.3.4. In particular, the value of *k* is negligible with respect to the term O(mn). Therefore, the total cost is

(13) $$T_{GeometryBased}=D\times O\left(mnMN\right)=O\left(MN\right).$$

**Figure 7:**
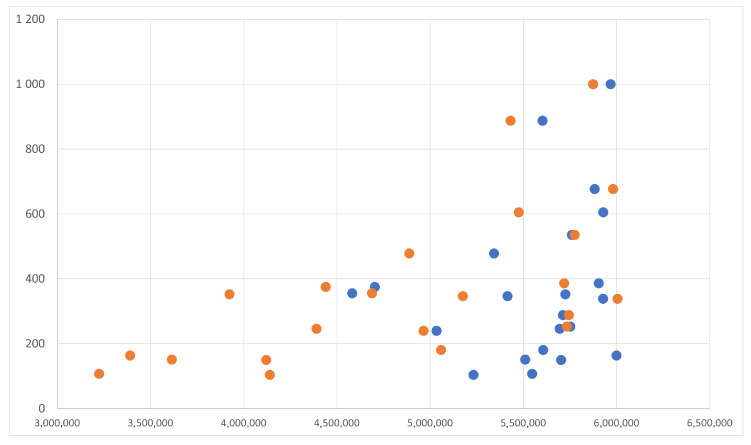
M×N (x-axis) versus computation time in seconds (y-axis). The orange dots takes into account when the uniform blocks are ignored from the procedure.

Since the enhancing procedure is the same for both the NCC-based and the geometry-based approaches and its computational cost is lower than the matching cost, it has not been considered in the above detailed computational analysis. Indeed, the overall cost of the enhancing procedure is linear, hence it can be simply added to the total cost of the matching step, which has an higher degree. Therefore, is possible to consider just the total costs of the compared matching procedures for their computational evaluation. The above analysis shows that the geometry-based reconstruction improves the efficiency by reducing the order of growth from quadratic (i.e., $O(M^{2}N^{2})$) to linear (i.e., O(MN)). Figure 7 shows the distribution of the computation time (in seconds) with respect to the image resolution (i.e., M×N). The blue dots represent the pairs (M×N, time), whereas the orange dots represent the same data after subtracting from M×N the number of pixels not processed due to the uniform block check. Is possible to observe that the latter set of points (i.e., orange dots) are distributed linearly with respect to the x-axis (i.e., M×N). The experiments have been performed on a set of images from the Middlebury dataset 2014 (i.e., the one with the highest resolution images).

## 4. Experiments

The proposed method has been successfully evaluated in [20] with high resolution images taken from real and challenging cases such as flowers compositions, natural scenes, animals, buildings, etc. The obtained results are reported in Table 1 considering the achieved bit-rate saving and lossy. In particular, the dataset includes 23 stereoscopic MPO images compliant with the [1] standard at different resolutions (1440×1080, 1620×1080, 1444×1080, 1924×1080, or 1920×1080). The JPEG quality factor has been set equal to 85 to encode the high quality image, whereas for the encoding of the low quality one it has been set to 65 or 70, by using standard quantization tables.

The achieved results show compression gain in terms of total bit-rate, while the quality loss is measured considering the Peak Signal to Noise Ratio (PSNR) measure. In particular, for each MPO image and for each value of JPEG compression factor used to compress the low quality image (i.e., 65 or 70) Table 1 reports the dimensions of the blocks used in decoding (second column), and the lossy (in terms of dB) computed on the reconstructed image obtained after the enhancing procedure. The bitrate saving is computed by comparing the space needed to encode the low quality image with the space needed to encode the same image using a quality factor equal to 85 (third and fifth columns). Note that the size of the blocks has been chosen ad hoc for each stereopair, depending on the resolution of the original images. Experiments show interesting bit-rate values with very low lossy. However the considered dataset only includes 23 stereopairs. In the proposed experiments, we considered a pool of well-known standard stereoscopic datasets built and publicly shared. Since 2001, the Middlebury Stereo Datasets have been considered to evaluate several stereoscopic algorithms, including stereoscopic image compression approaches. In the experiments here presented, we considered 60 stereopairs from all the Middlebury Stereo Dataset versions published between 2001 and 2014. Indeed, there are several versions that differ by the resolution of images and the employed acquisition technique. In particular, we considered:

- Five datasets of piecewise planar scenes of Middlebury-scenes 2001 [30];
- Two datasets of Middlebury-scenes 2003 [24];
- Nine datasets of Middlebury-scenes 2005 [31];
- Twenty-one datasets of Middlebury-scenes 2006 [32];
- Twenty-three datasets of high-resolution scenes of Middlebury-scenes 2014 [33].

The dimensions and variety of stereopair used on the proposed experiments allow us to perform a large evaluation of the pipeline on a standard and well-known benchmark set of stereopair images, as well as compare the performances of the pipeline on input of different resolutions. The approach has been evaluated by varying either the local feature detection and the fundamental matrix estimation algorithms, considering the SIFT or Harrys keypoints detection to define the initial set of image point correspondences and the 8-points algorithm or the Least Median of Squares optimization for the fundamental matrix estimation. As expected, the SIFT algorithm provides an high amount of correspondences with the presence of some outliers. The Harrys keypoints method detects a lower number of correspondences but with a lower outlier presence rate. As explained in Section 3.3.3, the selection of the best approach to estimate the fundamental matrix depends on the number and the quality of the available point correspondences between the two image views.

Due to space reasons, this paper only includes the results obtained on the Middlebury-scenes 2014 [33] and on the Middlebury-scenes 2006 [32] datasets. The complete results achieved on all the 60 considered stereopairs are detailed in the Supplementary Materials. In particular, the results of the experiments on the Middlebury-scenes 2014 dataset are shown in Table 2 (low quality factor 65) and Table 3 (low quality factor 70), whereas the results achieved on the Middlebury-scenes 2006 dataset are reported in Table 4 and Table 5. The achieved results confirmed that the adaptive approach allows to obtain high performances in terms of image quality, while requiring very low computational efforts. Several experiments obtained a reconstruction loss lower than $10^{-4}$ dB. By observing the experimental results, there is not a setting that strongly outperforms the others. However, considering the average performances in terms of lossy and computational time (reported on the last row of each Table), the methods based on the Least Median of Squares optimization to infer the fundamental matrix performs better in terms of lossy, whereas the methods based on the 8-points algorithm shown slightly better time performances.

### 4.1. Comparative Evaluation

The approaches in [11,13,14] have a pipeline similar to those used in the MPO standard [1], with the main difference in the encoding/decoding transform that is applied after quantization, namely curvelets, arithmetic encoding, and Huffman encoding, respectively. Such an encoding-decoding pipeline is independent from the stereopair acquisition settings. Those approaches have been evaluated on the popular LIVE 3D image quality database [34] of the University of Texas (The LIVE 3D image quality database is available at http://live.ece.utexas.edu/research/quality/live_3dimage_phase1.html (accessed on 21 June 2021) In this paragraph, we report the comparative evaluation of the adaptive approach [20] with respect to [11,13,14] considering the same evaluation settings. Table 6 shows the detailed results of the adaptive method applied on the LIVE 3D dataset, considering all the experimental settings involved in our benchmark. Note that we reported the PSNR for the right image only, indeed, all the experimental settings apply the same encoding/decoding pipeline to the left image, whereas a specific decoding process is applied on the right view. The PSNR of the left view is the same, regardless the experimental setting and is equal to 38.38744. Table 7 shows the comparison between the adaptive approach and the methods in [11,13,14]. Moreover, we also reported the performances obtained by applying the Standard MPO approach [1]. It is possible to observe that the adaptive method significantly outperforms the other approaches in terms of Compression Ratio. Regarding the PSNR measure, the Huffman coding proposed in [14] achieved the best results, with corresponding very low performances in terms of Compression Ratio (CR).

### 4.2. Subjective Assessment

**Figure 8:**
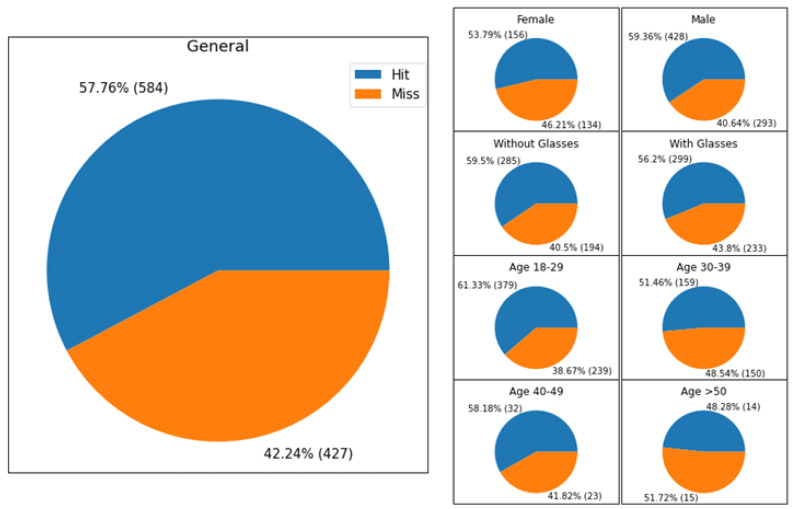
Detailed performances of hit/miss obtained during the subjective assessment.

While the adaptive method is the best in terms of CR, in order to assess the perceived quality of the images obtained by the adaptive method after reconstruction, we set up a subjective quality assessment experiment, described in this paragraph. In addition to the quality evaluation of the adaptive approach, in terms of bitrate saving, PSNR and computational time reported above, we also set up a subjective assessment of the reconstructed images’ quality. The experiments involved 116 people selected by considering variability among gender, age, and people with eyesight problems. In particular, each participant indicated if he/she uses corrective lenses or glasses, their gender, and their age, by selecting a set of age ranges (18–29, 30–39, 40–49, 50–59 or >60). Since we involved only a small number of people aged in the range 50–59 (i.e., 10), we merged the last two age ranges, creating the new category of people aged more than 50. During the acquisition session, each user was required to evaluate the quality of two images, one image is the right view of a stereopair at the original quality, whereas the other one is the same view after the compression and reconstruction pipeline described in this paper. Each user evaluated 10 image pairs randomly selected from the Middlebury datasets, and the position of the low quality image (left or right) is also selected randomly. We counted the number of times the users correctly selected the low quality image (hit) and the times the users selected the other ones (miss). Table 8 shows the percentage of hit and miss considering the different user categories. The general and detailed hit/miss percentage can be also observed in the pie charts shown in Figure 8. From the results is possible to observe that the involved participants were unable to distinguish the reconstructed image than the original one. This result is invariant considering also marginalizing the results by gender, age, or the presence of eyesight problems. We also performed an independence $Chi^{2}$ test, which confirmed the independence between the considered categories and the capability of guessing the right image. The test has been repeated considering several values of significance (from 0.01 to 0.05), in which the null hypothesis H${}_{0}$ (i.e., independence) were accepted with test confidence always higher than 99%.

## 5. Conclusions and Future Works

In this paper, an overview on stereoscopic image compression is first presented, with a focus on the problem of standardization of the compressed images. Then, an adaptive stereoscopic image compression approach first presented in [19] and further extended in [20] has been investigated and detailed. In particular, the advances in the matching strategy of the approach under analysis are presented. Then, the paper presents an evaluation of the adaptive stereo compression method under different settings, taking into account the compressed image quality, two different optimization methods, and two keypoints extraction techniques. The 16 resulting evaluation settings have been used to benchmark the compression method on the five Middlebury datasets published between 2001 and 2014, which represent an established standard for the objective assessment of algorithms in the field of stereoscopy. The results shown that the method is able to obtain high compressed stereoscopic images, while maintaining the visual quality, without requiring additional storing payload and allowing the usage of a file format conform with [1] specifications. In this benchmarking, we also evaluated the percepted quality of the reconstructed images by involving a large number of participants that were required to select the highest quality image between the two proposed, future extensions of this approach can evaluate the performances considering more advanced and specific Human Visual System based quality metrics [35], to properly assess to what extent we can compress the low quality image while maintaining high quality in the 3D perception of the scene. Extended experiments could be performed to significantly lower the quality of the images, and observing the effects (e.g., blocking artefacts) of using image blocks with resolution dimensions that are powers of two (8 × 8, 16 × 16, 32 × 32, etc.). Moreover, studies on stereoscopic image compression can be also performed on new applications and domains in which stereoscopy is being applied [36]. 

## Figures and Tables

**Table 1:** Results obtained in the first experiments reported in [19,20].

		Low Quality 65		Low Quality 70	
MPO Image	N × M	Lossy (dB)	Bit-Rate Saving	Lossy (dB)	Bit-Rate Saving
Flowers1	360 × 360	2.17	40.70%	1.65	34.60%
Flowers2	360 × 481	2	40.60%	1.32	34.50%
Flowers3	360 × 481	2.76	48.70%	2.76	41.70%
Castle	360 × 481	2.62	38.30%	2.18	32.50%
Dorm	360 × 360	2.73	37.10%	2.64	31.10%
Pelion	360 × 481	2.34	37.60%	2.34	31.80%
Hallway	360 × 482	2.33	37.60%	2.33	32.10%
Statue	360 × 483	2.59	41.90%	2.6	35.70%
Library	360 × 270	1.98	38.70%	1.71	32.70%
Hall	360 × 360	1.66	41.10%	1.4	34.90%
Garden Bridge	360 × 360	2.11	39.50%	1.82	33.50%
Autumn1	360 × 361	2.73	35.30%	2.53	29.80%
Autumn2	360 × 361	2.6	36.40%	2.4	30.60%
Autumn3	360 × 361	2.38	37.00%	2.15	31.20%
Autumn4	360 × 361	2.65	36.10%	2.44	30.40%
Animals1	360 × 240	2.16	38.80%	2.16	32.80%
Animals2	360 × 240	2.47	37.00%	2.18	31.30%
Cube	360 × 360	2.33	39.30%	2	33.30%
Covered	360 × 360	1.88	39.20%	1.73	33.40%
Garden	360 × 360	2.41	38.50%	2.15	32.50%
Snow	360 × 481	2.62	36.80%	2.45	31.20%
Tree	360 × 360	2.69	37.40%	2.52	31.40%
Zoo	360 × 240	2.67	36.90%	2.33	31.10%

**Table 2:** Results on the Middlebury-scenes 2014 dataset, considering a low quality of 65.

	Stereopair	Size	# of Blocks	Method								
				Low Quality 65								
				Bitrate Saving (%)	LmedS				Norm8Points			
					Harris		SIFT		Harris		SIFT	
ID					Lossy (dB)	Time (s)	Lossy (dB)	Time (s)	Lossy (dB)	Time (s)	Lossy (dB)	Time (s)
1	Adirondack-perfect	1988 × 2880	3.479	43	0.58	933	0.58	252	<$10^{-4}$	304	1.59	352
2	Backpack-perfect	2016 × 2940	3.600	39	1.32	846	<$10^{-4}$	684	1.32	239	1.33	339
3	Bicycle1-perfect	2008 × 2988	3.577	37	1.59	137	1.59	622	1.59	395	1.59	164
4	Cable-perfect	1984 × 2796	3.381	41	1.59	519	1.59	372	1.59	121	2.18	1.070
5	Classroom1-perfect	1920 × 3000	3.478	42	1.00	174	1.00	190	1.00	124	1.00	535
6	Couch-perfect	1992 × 2300	2.793	42	1.59	266	2.00	339	2.59	1.068	2.01	356
7	Flowers-perfect	1980 × 2880	3.479	38	2.17	306	2.17	378	2.59	1.197	2.59	1.503
8	Jadeplant-perfect	1988 × 2632	3.185	40	1.74	346	2.32	632	2.91	924	2.92	1.040
9	Mask-perfect	2008 × 2792	3.381	40	2.74	140	1.41	600	1.83	377	2.75	1.813
10	Motorcycle-perfect	2000 × 2964	3.577	38	1.74	218	1.74	525	1.74	303	2.07	605
11	Piano-perfect	1920 × 2820	3.243	40	1.74	170	1.74	304	2.06	178	2.07	347
12	Pipes-perfect	1924 × 2960	3.431	38	2.00	752	2.91	800	2.00	156	2.01	246
13	Playroom-perfect	1908 × 2800	3.243	38	2.32	349	2.33	687	2.32	265	2.32	478
14	Playtable-perfect	1848 × 2724	3.082	38	2.00	133	0.68	369	1.81	325	0.68	240
15	Recycle-perfect	1924 × 2864	3.290	42	<$10^{-4}$	154	<$10^{-4}$	230	<$10^{-4}$	92	<$10^{-4}$	151
16	Shelves-perfect	2000 × 2952	3.577	41	1.41	880	1.00	480	2.00	1.298	1.00	386
17	Shopvac-perfect	1996 × 2356	2.842	42	<$10^{-4}$	241	<$10^{-4}$	365	<$10^{-4}$	396	<$10^{-4}$	375
18	Sticks-perfect	2008 × 2864	3.430	38	2.42	164	1.26	351	2.23	778	1.49	253
20	Sword1-perfect	2020 × 2912	3.600	39	1.49	978	0.58	572	1.49	208	1.49	677
21	Sword2-perfect	2000 × 2856	3.430	42	2.59	156	1.00	378	1.00	748	1.00	288
22	Umbrella-perfect	2016 × 2960	3.650	42	<$10^{-4}$	183	<$10^{-4}$	646	<$10^{-4}$	391	1.00	1.000
23	Vintage-perfect	1924 × 2912	3.384	36	2.17	201	2.17	189	2.59	257	2.59	887
			**Average**	40	1.55	375	1.28	453	1.58	461	1.62	596
			**St. Dev.**	2.02	0.81	296	0.86	179	0.91	367	0.82	446

**Table 3:** Results on the middlebury-scenes 2014 dataset, considering a low quality of 70.

	Stereopair	Size	# of Blocks	Method								
				Low Quality 70								
				Bitrate Saving (%)	LmedS				Norm8Points			
					Harris		SIFT		Harris		SIFT	
ID					Lossy (dB)	Time (s)	Lossy (dB)	Time (s)	Lossy (dB)	Time (s)	Lossy (dB)	Time (s)
1	Adirondack-perfect	1988 × 2880	3479	38	<$10^{-4}$	208	<$10^{-4}$	292	<$10^{-4}$	113	1.17	582
2	Backpack-perfect	2016 × 2940	3600	33	0.32	679	<$10^{-4}$	541	1.59	1.184	1.33	474
3	Bicycle1-perfect	2008 × 2988	3577	32	0.59	111	1.59	726	2.18	359	2.18	484
4	Cable-perfect	1984 × 2796	3381	36	2.18	882	2.18	674	0.59	183	0.59	201
5	Classroom1-perfect	1920 × 3000	3478	37	1.00	148	1.00	156	1.00	890	1.00	584
6	Couch-perfect	1992 × 2300	2793	36	0.59	181	0.59	279	2.01	473	2.01	350
7	Flowers-perfect	1980 × 2880	3479	33	1.17	851	1.17	395	1.17	444	1.17	385
8	Jadeplant-perfect	1988 × 2632	3185	34	1.74	441	1.74	848	2.01	489	2.92	1.414
9	Mask-perfect	2008 × 2792	3381	34	1.84	774	2.16	1.448	2.16	294	1.84	633
10	Motorcycle-perfect	2000 × 2964	3577	33	1.42	392	1.74	458	1.74	249	2.07	392
11	Piano-perfect	1920 × 2820	3243	35	1.74	222	2.07	708	1.74	210	2.07	240
12	Pipes-perfect	1924 × 2960	3431	33	2.01	671	2.33	1.346	2.01	255	2.01	268
13	Playroom-perfect	1908 × 2800	3243	33	2.07	543	2.07	772	2.07	138	2.33	413
14	Playtable-perfect	1848 × 2724	3082	32	0.90	966	0.68	168	0.68	207	0.90	340
15	Recycle-perfect	1924 × 2864	3290	37	<$10^{-4}$	184	<$10^{-4}$	191	1.00	95	1.00	446
16	Shelves-perfect	2000 × 2952	3577	36	<$10^{-4}$	289	<$10^{-4}$	307	0.59	525	2.01	1.088
17	Shopvac-perfect	1996 × 2356	2842	37	<$10^{-4}$	269	<$10^{-4}$	306	<$10^{-4}$	1003	<$10^{-4}$	315
18	Sticks-perfect	2008 × 2864	3430	32	0.26	226	0.68	333	1.27	253	1.27	409
20	Sword1-perfect	2020 × 2912	3600	33	0.91	1488	0.59	407	1.91	1.737	0.91	319
21	Sword2-perfect	2000 × 2856	3430	36	2.18	1696	1.00	546	1.00	1.196	1.00	855
22	Umbrella-perfect	2016 × 2960	3650	38	1.00	223	1.00	733	1.00	242	1.00	1.134
23	Vintage-perfect	1924 × 2912	3384	31	1.17	324	1.17	169	2.59	165	1.17	241
			**Average**	35	1.05	535	1.08	537	1.38	486	1.45	526
			**St. Dev.**	2.08	0.76	433	0.81	352	0.73	437	0.68	320

**Table 4:** Results on the Middlebury-scenes 2006 dataset, considering a low quality of 65.

	Stereopair	Size	# of Blocks	Method								
				Low Quality 65								
				Bitrate Saving (%)	LmedS				Norm8Points			
					Harris		SIFT		Harris		SIFT	
ID					Lossy (dB)	Time (sec)	Lossy (dB)	Time (sec)	Lossy (dB)	Time (sec)	Lossy (dB)	Time (sec)
1	Aloe	555 × 641	224	37	1.58	6	2.01	11	1.83	4	2.01	11
2	Baby1	555 × 620	224	40	0.87	4	1.24	6	1.65	26	1.24	6
3	Baby2	555 × 620	224	39	1.02	12	1.02	9	1.35	18	1.02	9
4	Baby3	555 × 656	238	40	1.61	36	1.35	13	1.02	6	1.35	13
5	Bowling1	555 × 626	224	39	1.00	10	1.59	5	1.91	27	1.59	5
6	Bowling2	555 × 665	238	39	1.81	9	2.27	15	1.81	13	2.27	15
7	Cloth1	555 × 626	224	36	1.52	11	1.77	6	1.77	11	1.77	6
8	Cloth2	555 × 650	224	38	1.75	22	1.65	9	1.85	20	1.65	9
9	Cloth3	555 × 626	224	38	1.85	9	1.85	15	2.18	24	1.85	15
10	Cloth4	555 × 650	224	37	2.18	27	1.84	8	1.91	10	1.84	8
11	Flowerpots	555 × 656	238	39	1.59	16	2.01	12	1.59	7	2.01	12
12	Lampshade1	555 × 650	224	39	3.18	14	3.18	13	1.91	9	3.18	13
13	Lampshade2	555 × 650	224	39	2.01	8	2.01	6	2.01	14	2.01	6
14	Midd1	555 × 698	252	36	1.49	5	2.42	31	1.49	8	2.42	31
15	Midd2	555 × 683	238	36	1.27	5	1.27	10	1.27	4	1.27	10
16	Monopoly	555 × 665	238	36	1.27	8	1.94	26	1.94	5	1.94	26
17	Plastic	555 × 635	224	34	0.42	9	0.42	13	0.42	7	0.42	13
18	Rocks1	555 × 638	224	38	1.65	8	2.40	13	1.65	15	2.40	13
19	Rocks2	555 × 638	224	38	1.65	9	2.05	8	2.05	5	2.05	8
20	Wood1	555 × 686	238	42	0.59	15	0.59	6	1.33	29	0.59	6
21	Wood2	555 × 653	224	40	1.42	31	0.42	8	<$10^{-4}$	6	0.42	8
			**Average**	38	1.53	13	1.68	12	1.57	13	1.68	12
			**St. Dev.**	1.68	0.59	8.73	0.69	6.50	0.54	8.11	0.69	6.50

**Table 5:** Results on the Middlebury-scenes 2006 dataset, considering a low quality of 70.

	Stereopair	Size	# of Blocks	Method								
				Low Quality 70								
				Bitrate Saving (%)	LmedS				Norm8Points			
					Harris		SIFT		Harris		SIFT	
ID					Lossy (dB)	Time (s)	Lossy (dB)	Time (s)	Lossy (dB)	Time (s)	Lossy (dB)	Time (s)
1	Aloe	555 × 641	224	32	1.26	9	1.45	18	1.52	8	1.52	8
2	Baby1	555 × 620	224	34	0.66	4	1.32	34	1.32	28	0.87	7
3	Baby2	555 × 620	224	33	1.61	18	1.61	32	1.61	9	1.02	10
4	Baby3	555 × 656	238	34	0.91	6	1.17	40	0.91	16	0.91	11
5	Bowling1	555 × 626	224	33	1.00	10	1.00	26	1.91	16	1.00	5
6	Bowling2	555 × 665	238	33	2.27	30	1.40	14	1.40	7	2.08	8
7	Cloth1	555 × 626	224	31	1.83	28	1.47	29	1.47	9	1.71	13
8	Cloth2	555 × 650	224	32	1.32	13	1.21	15	1.75	10	1.65	7
9	Cloth3	555 × 626	224	32	1.50	10	1.50	16	1.69	6	1.50	10
10	Cloth4	555 × 650	224	31	2.05	25	1.49	13	1.49	10	1.78	8
11	Flowerpots	555 × 656	238	34	1.59	9	1.59	22	2.01	16	2.42	19
12	Lampshade1	555 × 650	224	33	2.33	11	2.33	14	2.59	26	2.33	8
13	Lampshade2	555 × 650	224	34	2.01	8	2.01	8	2.01	8	2.33	30
14	Midd1	555 × 698	252	31	1.27	11	1.27	11	1.27	8	1.49	9
15	Midd2	555 × 683	238	31	0.68	4	0.68	14	0.68	4	1.49	29
16	Monopoly	555 × 665	238	30	1.10	5	1.94	29	1.27	15	1.94	20
17	Plastic	555 × 635	224	29	1.42	22	0.42	6	0.42	18	0.42	8
18	Rocks1	555 × 638	224	32	2.27	33	1.33	9	2.40	37	1.65	7
19	Rocks2	555 × 638	224	32	1.50	9	0.91	6	1.65	9	1.65	6
20	Wood1	555 × 686	238	35	0.56	16	<$10^{-4}$	12	1.33	29	<$10^{-4}$	8
21	Wood2	555 × 653	224	33	1.42	25	0.42	10	<$10^{-4}$	6	1.42	22
			**Average**	32	1.47	15	1.26	18	1.46	14	1.48	12
			**St. Dev.**	1.45	0.53	8.98	0.55	9.89	0.61	9.09	0.62	7.40

**Table 6:** Performance of the [20] method applied on the LIVE 3D dataset [34].

	Lossy (dB)	Bitrate Saving	Time (s)	CR	PSNR Right
LmedSHarris70	1.4893	34.1709	2.0620	14.1925	36.6341
LmedSIFT70	1.4690	34.1709	1.6780	14.1925	36.6544
Norm8PointsHarris70	1.6734	34.1709	3.9440	14.1925	36.4500
Norm8PointSIFT70	1.4604	34.1709	1.5960	14.1925	36.6631
LmedSHarris65	1.6804	40.3686	1.1460	14.7500	36.4430
LmedSIFT65	1.6025	40.3686	4.3960	14.7500	36.5209
Norm8PointsHarris65	1.7414	40.3686	3.4940	14.7500	36.3820
Norm8PointSIFT65	1.6379	40.3686	1.0060	14.7500	36.4856

**Table 7:** Compression Ratio (CR) and average PSNR of the evaluated methods on the LIVE 3D dataset [34].

Method	CR	PSNR
Adaptive [20]	14.4713	37.4583
Standard MPO	11.6094	38.2526
Lossy SICCAC [11]	8.6400	41.5831
Lossy S2ICAC [13]	6.3600	33.9400
SICHC [14]	4.4271	49.1446
Lossless SICCAC [11]	4.1488	41.7359
Lossless S2ICAC [13]	3.6100	34.885

**Table 8:** Hit and miss collected during the subjective assessment experiments.

	Hit	Miss
Female	0.537931	0.462069
Male	0.593620	0.406380
Without Glasses	0.594990	0.405010
With Glasses	0.562030	0.437970
Age 18–29	0.613269	0.386731
Age 30–39	0.514563	0.485437
Age 40–49	0.581818	0.418182
Age > 50	0.482759	0.517241

## Data Availability

Data used in this paper are public.

## Supplementary Materials

The following are available online at https://www.mdpi.com/article/10.3390/jimaging7080160/s1, Table S1: Results on Middlebury-scenes 2014 dataset, considering a low quality of 65, Table S2: Results on Middlebury-scenes 2014 dataset, considering a low quality of 70, Table S3: Results on Middlebury-scenes 2006 dataset, considering a low quality of 65, Table S4: Results on Middlebury-scenes 2006 dataset, considering a low quality of 70, Table S5: Results on Middlebury-scenes 2005 dataset, considering a low quality of 65, Table S6: Results on Middleburyscenes 2005 dataset, considering a low quality of 70, Table S7: Results on Middlebury-scenes 2003 dataset, considering a low quality of 65, Table S8: Results on Middlebury-scenes 2003 dataset, considering a low quality of 70, Table S9: Results on Middlebury-scenes 2001 dataset, considering a low quality of 65 and Table S10: Results on Middlebury-scenes 2001 dataset, considering a low quality of 70.
