# Supplementary material for: A Benchmark Evaluation of Adaptive Image Compression for Multi Picture Object Stereoscopic Images
Source: J Imaging. 2021 Aug 23;7(8):160. doi: 10.3390/jimaging7080160 (PMC8404914; doi:10.3390/jimaging7080160)
Supplement: Supplementary file 1 [file jimaging-07-00160-s001.zip › Supplementary Material rev2.pdf]

Supplementary Material: *A Benchmark Evaluation of Adaptive Image Compression for Multi Picture Object Stereoscopic Images*

This document details the experimental results related to the paper entitled "*A Benchmark Evaluation of Adaptive Image Compression for Multi Picture Object Stereoscopic Images*". In the experiments here reported, the proposed method has been evaluated on 60 stereopairs from all the Middlebury Stereo Dataset versions published between 2001 and 2014, considering a low-quality factor equal to 65 or 70.

Table S1. Results on the Middlebury-scenes 2014 dataset, considering a low quality of 65.

ID	Stereopair	Size	# of Blocks	Method								
				Low Quality 65								
				Bitrate Saving (%)	LmedS				Norm8Points			
					Harris		SIFT		Harris		SIFT	
					Lossy (dB)	Time (s)	Lossy (dB)	Time (s)	Lossy (dB)	Time (s)	Lossy (dB)	Time (s)
1	Adirondack-perfect	1988 × 2880	3.479	43	0.58	933	0.58	252	$<10^{-4}$	304	1.59	352
2	Backpack-perfect	2016 × 2940	3.600	39	1.32	846	$<10^{-4}$	684	1.32	239	1.33	339
3	Bicycle1-perfect	2008 × 2988	3.577	37	1.59	137	1.59	622	1.59	395	1.59	164
4	Cable-perfect	1984 × 2796	3.381	41	1.59	519	1.59	372	1.59	121	2.18	1.070
5	Classroom1-perfect	1920 × 3000	3.478	42	1.00	174	1.00	190	1.00	124	1.00	535
6	Couch-perfect	1992 × 2300	2.793	42	1.59	266	2.00	339	2.59	1.068	2.01	356
7	Flowers-perfect	1980 × 2880	3.479	38	2.17	306	2.17	378	2.59	1.197	2.59	1.503
8	Jadeplant-perfect	1988 × 2632	3.185	40	1.74	346	2.32	632	2.91	924	2.92	1.040
9	Mask-perfect	2008 × 2792	3.381	40	2.74	140	1.41	600	1.83	377	2.75	1.813
10	Motorcycle-perfect	2000 × 2964	3.577	38	1.74	218	1.74	525	1.74	303	2.07	605
11	Piano-perfect	1920 × 2820	3.243	40	1.74	170	1.74	304	2.06	178	2.07	347
12	Pipes-perfect	1924 × 2960	3.431	38	2.00	752	2.91	800	2.00	156	2.01	246
13	Playroom-perfect	1908 × 2800	3.243	38	2.32	349	2.33	687	2.32	265	2.32	478
14	Playtable-perfect	1848 × 2724	3.082	38	2.00	133	0.68	369	1.81	325	0.68	240
15	Recycle-perfect	1924 × 2864	3.290	42	$<10^{-4}$	154	$<10^{-4}$	230	$<10^{-4}$	92	$<10^{-4}$	151
16	Shelves-perfect	2000 × 2952	3.577	41	1.41	880	1.00	480	2.00	1.298	1.00	386
17	Shopvac-perfect	1996 × 2356	2.842	42	$<10^{-4}$	241	$<10^{-4}$	365	$<10^{-4}$	396	$<10^{-4}$	375
18	Sticks-perfect	2008 × 2864	3.430	38	2.42	164	1.26	351	2.23	778	1.49	253
20	Sword1-perfect	2020 × 2912	3.600	39	1.49	978	0.58	572	1.49	208	1.49	677
21	Sword2-perfect	2000 × 2856	3.430	42	2.59	156	1.00	378	1.00	748	1.00	288
22	Umbrella-perfect	2016 × 2960	3.650	42	$<10^{-4}$	183	$<10^{-4}$	646	$<10^{-4}$	391	1.00	1.000
23	Vintage-perfect	1924 × 2912	3.384	36	2.17	201	2.17	189	2.59	257	2.59	887
			**Average**	40	1.55	375	1.28	453	1.58	461	1.62	596
			**St. Dev.**	2.02	0.81	296	0.86	179	0.91	367	0.82	446

Table S2. Results on the middlebury-scenes 2014 dataset, considering a low quality of 70.

ID	Stereopair	Size	# of Blocks	Method								
				Low Quality 70								
				Bitrate Saving (%)	LmedS				Norm8Points			
					Harris		SIFT		Harris		SIFT	
					Lossy (dB)	Time (s)	Lossy (dB)	Time (s)	Lossy (dB)	Time (s)	Lossy (dB)	Time (s)
1	Adirondack-perfect	1988 × 2880	3479	38	$<10^{-4}$	208	$<10^{-4}$	292	$<10^{-4}$	113	1.17	582
2	Backpack-perfect	2016 × 2940	3600	33	0.32	679	$<10^{-4}$	541	1.59	1.184	1.33	474
3	Bicycle1-perfect	2008 × 2988	3577	32	0.59	111	1.59	726	2.18	359	2.18	484
4	Cable-perfect	1984 × 2796	3381	36	2.18	882	2.18	674	0.59	183	0.59	201
5	Classroom1-perfect	1920 × 3000	3478	37	1.00	148	1.00	156	1.00	890	1.00	584
6	Couch-perfect	1992 × 2300	2793	36	0.59	181	0.59	279	2.01	473	2.01	350
7	Flowers-perfect	1980 × 2880	3479	33	1.17	851	1.17	395	1.17	444	1.17	385
8	Jadeplant-perfect	1988 × 2632	3185	34	1.74	441	1.74	848	2.01	489	2.92	1.414
9	Mask-perfect	2008 × 2792	3381	34	1.84	774	2.16	1.448	2.16	294	1.84	633
10	Motorcycle-perfect	2000 × 2964	3577	33	1.42	392	1.74	458	1.74	249	2.07	392
11	Piano-perfect	1920 × 2820	3243	35	1.74	222	2.07	708	1.74	210	2.07	240
12	Pipes-perfect	1924 × 2960	3431	33	2.01	671	2.33	1.346	2.01	255	2.01	268
13	Playroom-perfect	1908 × 2800	3243	33	2.07	543	2.07	772	2.07	138	2.33	413
14	Playtable-perfect	1848 × 2724	3082	32	0.90	966	0.68	168	0.68	207	0.90	340
15	Recycle-perfect	1924 × 2864	3290	37	$<10^{-4}$	184	$<10^{-4}$	191	1.00	95	1.00	446
16	Shelves-perfect	2000 × 2952	3577	36	$<10^{-4}$	289	$<10^{-4}$	307	0.59	525	2.01	1.088
17	Shopvac-perfect	1996 × 2356	2842	37	$<10^{-4}$	269	$<10^{-4}$	306	$<10^{-4}$	1003	$<10^{-4}$	315
18	Sticks-perfect	2008 × 2864	3430	32	0.26	226	0.68	333	1.27	253	1.27	409
20	Sword1-perfect	2020 × 2912	3600	33	0.91	1488	0.59	407	1.91	1.737	0.91	319
21	Sword2-perfect	2000 × 2856	3430	36	2.18	1696	1.00	546	1.00	1.196	1.00	855
22	Umbrella-perfect	2016 × 2960	3650	38	1.00	223	1.00	733	1.00	242	1.00	1.134
23	Vintage-perfect	1924 × 2912	3384	31	1.17	324	1.17	169	2.59	165	1.17	241
			**Average**	35	1.05	535	1.08	537	1.38	486	1.45	526
			**St. Dev.**	2.08	0.76	433	0.81	352	0.73	437	0.68	320

Table S3. Results on the Middlebury-scenes 2006 dataset, considering a low quality of 65.

				Method								
				Low Quality 65								
	Stereopair	Size	# of Blocks		LmedS				Norm8Points			
				Bitrate Saving (%)	Harris		SIFT		Harris		SIFT	
ID					Lossy (dB)	Time (sec)	Lossy (dB)	Time (sec)	Lossy (dB)	Time (sec)	Lossy (dB)	Time (sec)
1	Aloe	555 × 641	224	37	1.58	6	2.01	11	1.83	4	2.01	11
2	Baby1	555 × 620	224	40	0.87	4	1.24	6	1.65	26	1.24	6
3	Baby2	555 × 620	224	39	1.02	12	1.02	9	1.35	18	1.02	9
4	Baby3	555 × 656	238	40	1.61	36	1.35	13	1.02	6	1.35	13
5	Bowling1	555 × 626	224	39	1.00	10	1.59	5	1.91	27	1.59	5
6	Bowling2	555 × 665	238	39	1.81	9	2.27	15	1.81	13	2.27	15
7	Cloth1	555 × 626	224	36	1.52	11	1.77	6	1.77	11	1.77	6
8	Cloth2	555 × 650	224	38	1.75	22	1.65	9	1.85	20	1.65	9
9	Cloth3	555 × 626	224	38	1.85	9	1.85	15	2.18	24	1.85	15
10	Cloth4	555 × 650	224	37	2.18	27	1.84	8	1.91	10	1.84	8
11	Flowerpots	555 × 656	238	39	1.59	16	2.01	12	1.59	7	2.01	12
12	Lampshade1	555 × 650	224	39	3.18	14	3.18	13	1.91	9	3.18	13
13	Lampshade2	555 × 650	224	39	2.01	8	2.01	6	2.01	14	2.01	6
14	Midd1	555 × 698	252	36	1.49	5	2.42	31	1.49	8	2.42	31
15	Midd2	555 × 683	238	36	1.27	5	1.27	10	1.27	4	1.27	10
16	Monopoly	555 × 665	238	36	1.27	8	1.94	26	1.94	5	1.94	26
17	Plastic	555 × 635	224	34	0.42	9	0.42	13	0.42	7	0.42	13
18	Rocks1	555 × 638	224	38	1.65	8	2.40	13	1.65	15	2.40	13
19	Rocks2	555 × 638	224	38	1.65	9	2.05	8	2.05	5	2.05	8
20	Wood1	555 × 686	238	42	0.59	15	0.59	6	1.33	29	0.59	6
21	Wood2	555 × 653	224	40	1.42	31	0.42	8	$<10^{-4}$	6	0.42	8
			Average	38	1.53	13	1.68	12	1.57	13	1.68	12
			St. Dev.	1.68	0.59	8.73	0.69	6.50	0.54	8.11	0.69	6.50

Table S4. Results on the Middlebury-scenes 2006 dataset, considering a low quality of 70.

				Method								
				Low Quality 70								
	Stereopair	Size	# of Blocks		LmedS				Norm8Points			
				Bitrate Saving (%)	Harris		SIFT		Harris		SIFT	
ID					Lossy (dB)	Time (s)	Lossy (dB)	Time (s)	Lossy (dB)	Time (s)	Lossy (dB)	Time (s)
1	Aloe	555 × 641	224	32	1.26	9	1.45	18	1.52	8	1.52	8
2	Baby1	555 × 620	224	34	0.66	4	1.32	34	1.32	28	0.87	7
3	Baby2	555 × 620	224	33	1.61	18	1.61	32	1.61	9	1.02	10
4	Baby3	555 × 656	238	34	0.91	6	1.17	40	0.91	16	0.91	11
5	Bowling1	555 × 626	224	33	1.00	10	1.00	26	1.91	16	1.00	5
6	Bowling2	555 × 665	238	33	2.27	30	1.40	14	1.40	7	2.08	8
7	Cloth1	555 × 626	224	31	1.83	28	1.47	29	1.47	9	1.71	13
8	Cloth2	555 × 650	224	32	1.32	13	1.21	15	1.75	10	1.65	7
9	Cloth3	555 × 626	224	32	1.50	10	1.50	16	1.69	6	1.50	10
10	Cloth4	555 × 650	224	31	2.05	25	1.49	13	1.49	10	1.78	8
11	Flowerpots	555 × 656	238	34	1.59	9	1.59	22	2.01	16	2.42	19
12	Lampshade1	555 × 650	224	33	2.33	11	2.33	14	2.59	26	2.33	8
13	Lampshade2	555 × 650	224	34	2.01	8	2.01	8	2.01	8	2.33	30
14	Midd1	555 × 698	252	31	1.27	11	1.27	11	1.27	8	1.49	9
15	Midd2	555 × 683	238	31	0.68	4	0.68	14	0.68	4	1.49	29
16	Monopoly	555 × 665	238	30	1.10	5	1.94	29	1.27	15	1.94	20
17	Plastic	555 × 635	224	29	1.42	22	0.42	6	0.42	18	0.42	8
18	Rocks1	555 × 638	224	32	2.27	33	1.33	9	2.40	37	1.65	7
19	Rocks2	555 × 638	224	32	1.50	9	0.91	6	1.65	9	1.65	6
20	Wood1	555 × 686	238	35	0.56	16	$<10^{-4}$	12	1.33	29	$<10^{-4}$	8
21	Wood2	555 × 653	224	33	1.42	25	0.42	10	$<10^{-4}$	6	1.42	22
			Average	32	1.47	15	1.26	18	1.46	14	1.48	12
			St. Dev.	1.45	0.53	8.98	0.55	9.89	0.61	9.09	0.62	7.40

Table S5. Results on the middlebury-scenes 2005 dataset, considering a low quality of 65.

				Method								
				Low Quality 65								
	Stereopair	Size	# of Blocks		LmedS				Norm8Points			
				Bitrate Saving (%)	Harris		SIFT		Harris		SIFT	
ID					Lossy (dB)	Time (sec)	Lossy (dB)	Time (sec)	Lossy (dB)	Time (sec)	Lossy (dB)	Time (sec)
1	Art	555X695	238	42	2,10	42	1,98	22	1,72	15	1,98	13
2	Books	555X695	238	15	2,01	15	2,28	23	2,28	11	2,61	37
3	Computer	555X665	238	13	1,69	13	1,69	8	1,95	7	1,95	12
4	Dolls	555X695	238	10	1,33	10	1,33	12	1,87	43	1,76	13
5	Drumsticks	555X695	238	13	2,01	13	2,01	10	2,12	7	2,12	13
6	Dwarves	555X695	238	13	1,59	13	1,59	11	1,59	16	1,59	10
7	Laundry	555X671	238	13	2,00	13	1,84	11	2,40	8	2,40	45
8	Moebius	555X695	238	18	1,62	18	1,62	12	1,62	9	2,17	26
9	Reindeer	555X671	238	9	1,84	9	1,64	12	1,84	6	1,84	8
			Average	16	1,80	16	1,78	14	1,93	13	2,05	20

Table S6. Results on the Middlebury-scenes 2005 dataset, considering a low quality of 70.

ID	Stereopair	Size	# of Blocks	Bitrate Saving (%)	Method							
					Low Quality 70							
					LmedS				Norm8Points			
					Harris		SIFT		Harris		SIFT	
					Lossy (dB)	Time (sec)	Lossy (dB)	Time (sec)	Lossy (dB)	Time (sec)	Lossy (dB)	Time (sec)
1	Art	555X695	238	32	1,39	23	1,84	33	1,98	30	1,39	14
2	Books	555X695	238	31	1,90	16	2,28	37	2,28	28	1,90	12
3	Computer	555X665	238	30	1,27	9	1,10	9	1,10	6	1,95	9
4	Dolls	555X695	238	32	1,76	25	1,23	15	1,76	30	1,33	12
5	Drumsticks	555X695	238	32	1,74	9	1,74	31	2,01	15	2,01	34
6	Dwarves	555X695	238	32	2,01	29	1,13	9	1,40	16	1,59	24
7	Laundry	555X671	238	30	1,69	11	1,69	11	2,00	17	2,13	42
8	Moebius	555X695	238	33	1,47	12	1,14	9	1,47	6	1,62	9
9	Reindeer	555X671	238	35	2,01	42	1,64	28	1,23	6	1,84	7
			Average	32	1,69	19	1,53	20	1,69	17	1,75	18

Table S7. Results on the Middlebury-scenes 2003 dataset, considering a low quality of 65.

ID	Stereopair	Size	# of Blocks	Bitrate Saving (%)	Method							
					Low Quality 65							
					LmedS				Norm8Points			
					Harris		SIFT		Harris		SIFT	
					Lossy (dB)	Time (sec)	Lossy (dB)	Time (sec)	Lossy (dB)	Time (sec)	Lossy (dB)	Time (sec)
1	cones	375X450	110	39	1,26	3	1,26	3	1,30	7	1,30	2
2	teddy	375X450	110	40	1,21	3	1,21	2	1,52	8	1,41	6
			Average	40	1,23	3	1,23	3	1,41	7	1,36	4

Table S8. Results on the Middlebury-scenes 2003 dataset, considering a low quality of 70.

ID	Stereopair	Size	# of Blocks	Bitrate Saving (%)	Method							
					Low Quality 70							
					LmedS				Norm8Points			
					Harris		SIFT		Harris		SIFT	
					Lossy (dB)	Time (sec)	Lossy (dB)	Time (sec)	Lossy (dB)	Time (sec)	Lossy (dB)	Time (sec)
1	cones	375X450	110	33	1,13	3	1,17	5	1,38	7	1,33	3
2	teddy	375X450	110	34	1,12	4	1,05	3	1,12	3	1,12	2
			Average	34	1,13	3	1,11	4	1,25	5	1,22	3

Table S9. Results on the Middlebury-scenes 2001 dataset, considering a low quality of 65.

ID	Stereopair	Size	# of Blocks	Bitrate Saving (%)	Method							
					Low Quality 65							
					LmedS				Norm8Points			
					Harris		SIFT		Harris		SIFT	
					Lossy (dB)	Time (sec)	Lossy (dB)	Time (sec)	Lossy (dB)	Time (sec)	Lossy (dB)	Time (sec)
1	barn1	381X432	110	40	1,34	7	1,17	2	1,17	3	1,34	2
2	barn2	381X430	110	42	0,98	1	0,98	2	0,98	3	0,98	1
3	bull	381X433	110	42	1,00	1	1,00	2	1,00	2	1,14	3
4	sawtooth	380X434	110	39	1,45	3	1,45	2	1,63	2	1,68	3
5	venus	383X434	110	40	1,49	7	1,27	5	1,21	3	1,27	2
			Average	40	1,25	4	1,17	3	1,20	2	1,28	2

Table S10. Results on the Middlebury-scenes 2001 dataset, considering a low quality of 70.

ID	Stereopair	Size	# of Blocks	Bitrate Saving (%)	Method							
					Low Quality 70							
					LmedS				Norm8Points			
					Harris		SIFT		Harris		SIFT	
					Lossy (dB)	Time (sec)	Lossy (dB)	Time (sec)	Lossy (dB)	Time (sec)	Lossy (dB)	Time (sec)
1	barn1	381X432	110	34	1,09	2	1,09	3	1,09	3	1,26	2
2	barn2	381X430	110	35	0,77	1	0,77	2	0,77	2	1,04	4
3	bull	381X433	110	35	0,91	3	1,00	3	0,91	3	1,00	2
4	sawtooth	380X434	110	33	1,34	3	1,34	2	1,86	8	1,58	4
5	venus	383X434	110	34	1,08	7	1,08	4	1,32	4	1,27	2
			Average	34	1,04	3	1,06	3	1,19	4	1,23	3
